# Blood perfusion in Hewes tarsoconjunctival flaps in pigs measured by laser speckle contrast imaging

**DOI:** 10.1016/j.jpra.2018.07.001

**Published:** 2018-07-29

**Authors:** Cu Dinh Ansson, Rafi Sheikh, Ulf Dahlstrand, Jenny Hult, Sandra Lindstedt, Malin Malmsjö

**Affiliations:** a Department of Clinical Sciences Lund, Skåne University Hospital, Lund University, Ögonklinik A, Kioskgatan 1B, SE-221 85 Lund*,* Sweden; b Lund University, Skåne University Hospital, Department of Clinical Sciences Lund, Cardiothoracic Surgery, Lund, Sweden

## Abstract

**Background:**

Hewes flap is a tarsoconjunctival eyelid flap, based at the lateral canthal tendon, and rotated and stretched to repair lateral defects in the lower eyelid commonly following tumor surgery. The aim of the present study was to monitor perfusion in a Hewes flap during reconstruction, which to the best of our knowledge, has not previously been done.

**Methods:**

A Hewes tarsoconjunctival eyelid flap was raised and the effects on blood perfusion of rotating the flaps by 90° and 180°, stretching the flaps with a force of 5 or 10 N, and repeated diathermic coagulation was monitored with laser speckle contrast imaging.

**Results:**

Rotating the flaps by 90° did not significantly affect perfusion, while further rotation to 180° reduced blood perfusion to 75% of the baseline value. When the tarsoconjunctival flaps were both rotated 90° and stretched with 5 N, the perfusion was reduced even further, to 63%. A further reduction in perfusion, to 36%, was seen when the higher force of 10 N was applied. Diathermy decreased blood perfusion to 56% after being applied once. Successive applications led to further decreases: 43%, 31%, and 15%, after the second, third and fourth applications.

**Conclusions:**

Perfusion in Hewes tarsoconjunctival flaps is affected by both rotation and stretching, but some perfusion is maintained despite these manipulations. Diathermy, however, has detrimental effects and should be avoided.

## Introduction

In 1976 Hewes et al. described a tarsoconjunctival flap that could be used to repair full-thickness lateral eyelid defect, e.g. after eyelid tumor excision or trauma, where the use of direct closure or Tenzel flap is not sufficient.^1^ The Hewes flap is a horizontal strip of tarsoconjunctival tissue, based at the lateral cantus, rotated around the long-axis and transposed from the upper eyelid to cover a defect on the lower eyelid. The advantage with the Hewes technique compared to Hughes procedure is the eye is not occluded for a period of several weeks.^2^

When the technique was first introduced, there were no adequate methods of measuring blood flow during reconstructive surgery, and the Hewes reconstruction was based on clinical trial and error.^1^ As the flap survived, it was concluded that this was a good reconstructive technique. In recent years, modern techniques have evolved for the measurement of blood flow.^3^, ^4^, ^5^ To the best of our knowledge, blood flow has not been monitored during the Hewes flap procedure. Laser speckle contrast imaging (LSCI) now enables non-invasive measurements of perfusion over the entire operating area, during the procedure.^6^, ^7^, ^8^, ^9^ The aim of this study was to monitor the blood flow in a Hewes tarsoconjunctival flap, using LSCI, in an experimental porcine model. The effects of the rotation and stretch needed to repair lateral defects in the lower eyelid commonly, as well as diathermy, were studied.

## Methods

### Ethics

The experimental protocol for this study was approved by the Ethics Committee for Animal Research at Lund University, Sweden.

### Surgical technique and experimental procedure

Anesthesia was performed as previously described.^10^ Anesthesia was performed in the same way in all animals. Blood pressure and pulse were monitored and kept within 120–140 / 80–100 mmHg and 60–80 bpm, respectively. Room temperature was kept constant at 20 °C.

**Figure 1:**
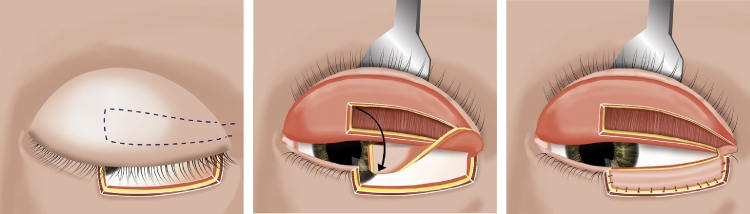
Illustration showing how the Hewes flap was raised. The upper tarsus was everted. A horizontal incision was made through the conjunctiva and Müller's muscle and a second parallel incision, superior to the attachment of the aponeurosis of the levator palpebrae superioris muscle, to create a 5 mm high and 30 mm long strip of the tarsoconjunctiva. The flap was then rotated and stretched for insertion in the lower eyelid to cover a defect, most commonly after tumor surgery.

A Hewes tarsoconjunctival eyelid flap was raised as follows: The upper tarsus was everted. A horizontal incision was made through the conjunctiva and Müller's muscle with a scalpel. A 5 mm horizontal strip of the tarsoconjunctiva was formed by a second parallel incision, superior to the attachment of the aponeurosis of the levator palpebrae superioris muscle. The flap was made 30 mm long. Sharp dissection freed the tarsus from the preaponeurotic space. The incision included the lateral canthal tendon and adjacent orbicularis oculi muscle to form the proximal end of the tarsoconjunctival flap. In this way, a tarsoconjunctival eyelid flap, based at the lateral canthal tendon, was created (Figure 1).

For the rotation and stretching experiments, the flaps were rotated manually, by 90° or 180°, using forceps. The effects of rotating the conjunctive flap by 90° and stretching it with a force of 5 N or 10 N were also investigated. A suture was attached to the tip of the flap which was then attached to a digital scale. The flap was manually extended by pulling on the scale until it showed 0.5 kg (a force of 5 N) or 1.0 kg (a force of 10 N).

In the diathermy experiments, diathermy was applied four times at a location approximately 5 mm distal from the base, using monopolar diathermy (7 W, Valleylab Force 2, Covidien, Medtronic, Minneapolis, MN, USA). The first application was at the upper edge of the base, the second at the lower edge of the base, and the third and fourth applications on the dorsal side of the base.

When the rotation and stretching and diathermy experiments had been concluded, the blood supply to the base of the conjunctival flap was occluded by applying sufficient pressure to occlude the blood flow to the flap. This was done to obtain a value for zero blood flow, as both laser-based techniques measure the blood flow in arbitrary units, rather than absolute units. Care was taken not to damage the tissue, and we also ensured that blood flow returned when the forceps were removed. Blood perfusion was measured in the tarsoconjunctival flap before mechanical manipulation, using LSCI, to obtain a baseline value. The effect of rotation/stretching and diathermy on blood perfusion was measured in the middle of the flap.

### Laser speckle contrast imaging

Tissue perfusion was measured with a PeriCam PSI NR System (Perimed AB, Stockholm, Sweden). This technique visualizes motion over an area of up to 24 × 24 cm. A camera records the changes in the laser speckle pattern at a rate of up to 100 images per second, with a spatial resolution of up 100 µm/pixel and the smallest possible measuring area is therefore 100 × 100 µm, which equals to 0.01 mm^2^.

### Calculations and statistics

The effects of rotation and stretching were studied in flaps from 16 eyelids in eight pigs. Diathermy was studied in flaps from 12 eyelids in six pigs. The diathermy experiments were not performed on the first two pigs for practical reasons. Blood perfusion was expressed as a percent (median values) after normalization to the baseline and occlusion values, which were set to 100% and 0%, respectively. Statistical analysis was performed using Friedman's test with Dunn's multiple comparison test. Significance was defined as *p* < 0.05 (*), *p* < 0.01 (**), *p* < 0.001 (***), and *p* > 0.05 (not significant, n.s.). Calculations and statistical analysis were performed using GraphPad Prism 7.2 (GraphPad Software Inc., San Diego, CA, USA).

## Results

**Figure 2:**
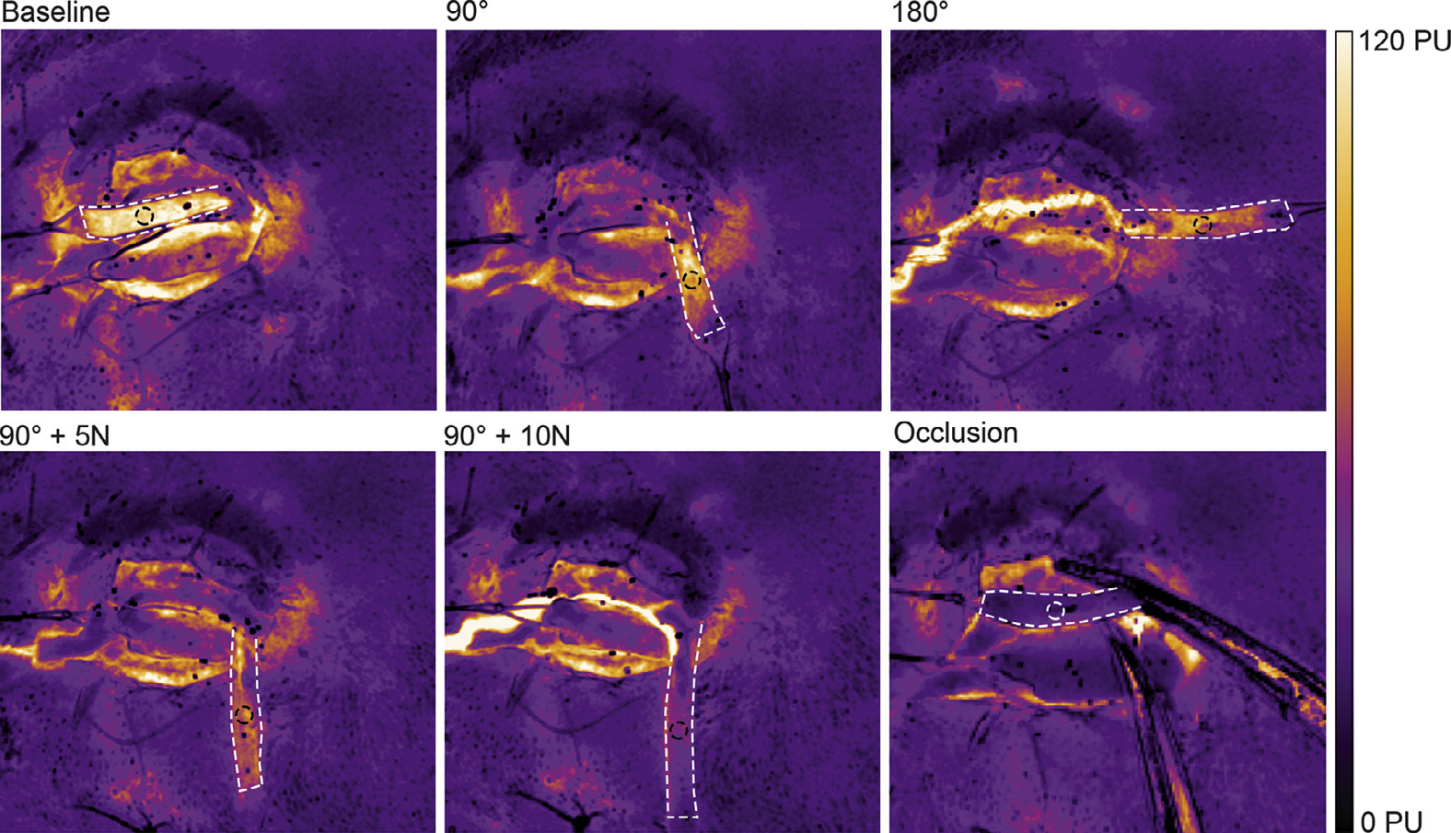
Representative LSCI images showing blood perfusion in Hewes flaps rotated 90^o^ and 180^o^, with 5 and 10 N stretching forces, and finally occlusion of the blood flow. The color represent perfusion in which white and yellow is high perfusion and purple and black is less perfusion (see scale bar).The effect of rotation/stretching on blood perfusion was measured in the middle of the flap (circle). Note the gradual decrease in perfusion during the manipulations.

Rotating the flaps by 90° had no significant effect on perfusion, while further rotation to 180° reduced the perfusion to 75% of the baseline value (*p* = 0.048). When a stretching force of 5 N was applied to the already rotated flap (90°), the perfusion decreased to 63% of the baseline value (*p* = 0.022). Increasing the stretching force to 10 N, caused the perfusion to decrease further, to 36% of the baseline value (*p* < 0.001). Figure 2 shows a representative example, and detailed results are given in Figure 4a

**Figure 3:**
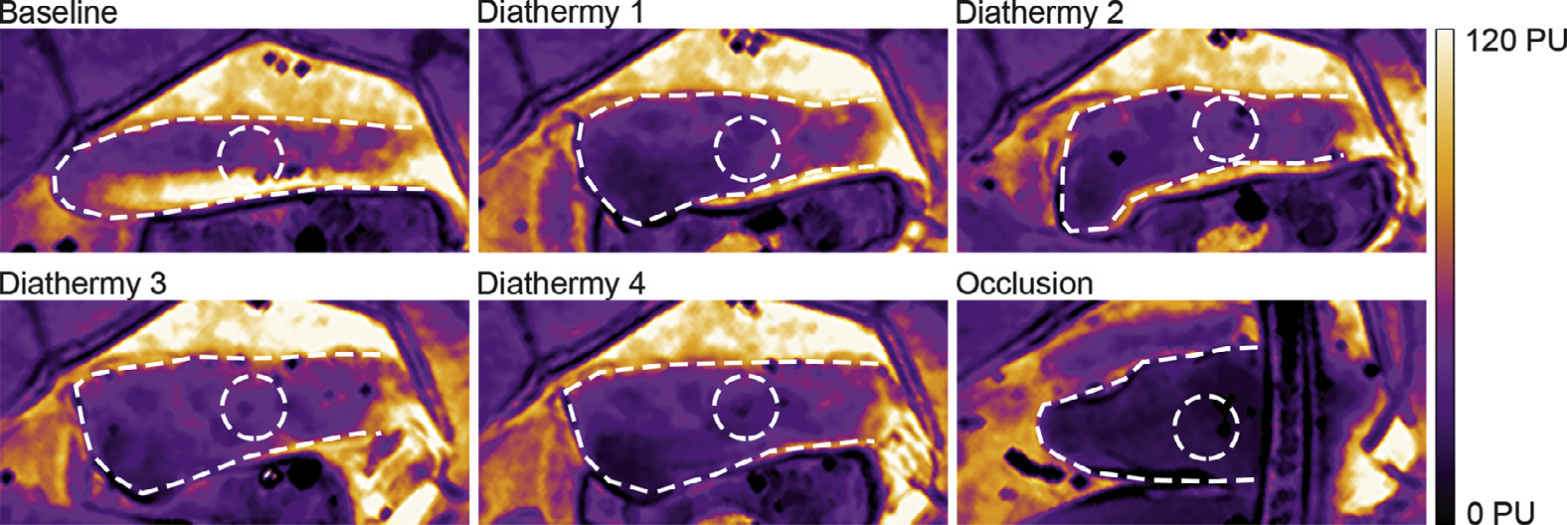
Detailed LSCI images showing blood perfusion in Hewes flaps before and after repeated diathermy, and finally occlusion of the blood flow. The color represent perfusion in which white and yellow is high perfusion and purple and black is less perfusion (see scale bar). The effect of diathermy on blood perfusion was measured in the middle of the flap (circle).

**Figure 4:**
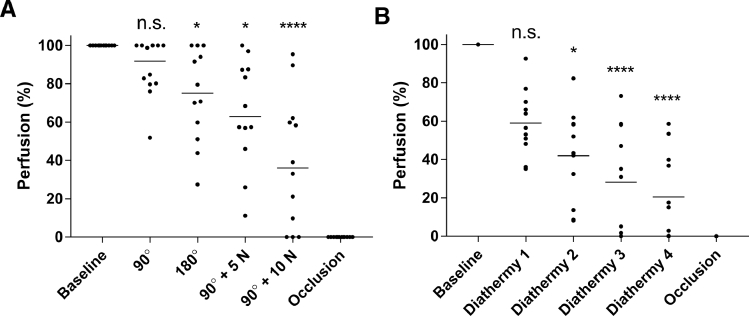
Scatter plots showing: (a) blood perfusion in flaps rotated 90^o^ and 180^o^, and stretched with forces of 5 and 10 N, and (b) the effect of applying diathermic coagulation four times/at four different locations.

Diathermic coagulation had detrimental effects on perfusion. The blood perfusion decreased after each of the four applications to the base of the eyelid flap, as can be seen in Figure 3. After the first application, the perfusion was reduced to 56% of the baseline value (*p* = 0.319). Successive applications led to further decreases, to 43% (*p*=0.012), 31% (*p* < 0.0001), and 15% (*p* < 0.0001) after the second, third and fourth applications (Figure 4b).

## Discussion

Full-thickness lower eyelid defects after tumor surgery are often repaired with a tarsoconjunctival flap, i.e. a modified Hughes flap or a Hewes flap.^1^, ^2^^,^^11^ Recent studies on blood perfusion in modified Hughes flaps have shown that blood flow in the flap decreased gradually during dissection and advancement of the flap.^12^ At the time when the flap was sutured into place, there was virtually no blood flow in the tissue.^12^ In this study we wanted to monitor the perfusion of Hewes flap during reconstruction, in detail. According to Hewes et al., the flap should be rotated 180° around the long axis.^1^ The results of this study showed that rotation by 180° reduced the perfusion to 75% of the baseline. We obtained similar results in a previous study on random skin flaps in pigs, showing a reduction in perfusion to 54% when rotated to 90°.^10^ It should be borne in mind when comparing these results that the conjunctiva is more richly vascularized than skin.

In the Hewes procedure, the apex of the tarsoconjunctival flap should be 2 to 3 mm temporal to that of the surgical defect; in other words, the flap needs to be stretched by 2–3 mm to cover the defect. The tension applied to the flap when it is sutured into place is intended to provide some lifting of the lower eyelid to prevent ectropion. The results of the present study showed that the combination of rotation and tension had considerable effects on perfusion, which may be alleviated by making the flap as long as the defect and thereby avoid stretching the flap.

In a previous study on random porcine skin flaps with a combination of 90° rotation and 3 N stretching we found that blood perfusion decreased by 78%.^10^ The present results are in line with those from this previous study, but the blood perfusion measured in the present study was higher. This could be due to the much higher degree of vascularization in conjunctival flaps than in random skin flaps. Reports in the literature suggest that the amount of stretching that is tolerated may vary at different locations in the body, but ranges from 1 N to as high as 16 N on the abdomen. Nevertheless, caution should be exercised when stretching a flap. Indeed, it has been shown that stretching increases the risk of necrosis in advancement flaps.^13^

Diathermy at the flap base seems to have detrimental effects on perfusion. After applying diathermy four times, the perfusion was only 15% of the baseline value. Similar results were reported in one of our recent publications on human eyelid skin flaps, where a dramatic decrease in blood perfusion to only 4% was observed after the second application of diathermy.^9^ Diathermy should be avoided as far as possible in the flap pedicle, to ensure adequate vascularization of the flap. If perfusion is completely obliterated, the flap will instead function as a free transplant, which may also survive. Indeed, it has been shown that there is little, if any, perfusion in the distal end of Hughes tarsoconjunctival flap^12^ and despite this, the Hughes flaps survives. Furthermore, in one of our recent reports we described a free full-thickness eyelid graft that healed well despite the absence of a vascularized flap, and this may be an alternative surgical technique in the future.^14^ The reason for the excellent healing despite the absence of a vascularized flap may be the high levels of oxygen and nutrients in the tear film, comparable to those in the blood.

One limitation of the present study is that blood perfusion was only measured immediately after manipulation of the flaps, and not over a longer time. Furthermore, the effects on long-term survival of the Hewes flaps were not studied. This study was carried out on pigs, and studies on humans are needed before any conclusions of clinical use can be drawn. Another limitation is the problem with movement artefacts. In this case this were concurred by keeping the flap as still as possible upon measured and the results were given as percent of baseline values, which presumably eliminates such confounding factors.

In conclusion, the laser speckle contrast imaging is a useful tool for monitoring the vascularity of small flaps. In a Hewes tarsoconjunctival flap, blood perfusion decreases significantly when conjunctival flaps were both rotated and stretched, although some perfusion remained. The use of diathermy on the flap should, however, be avoided since this has detrimental effects on the blood perfusion.
